# Role of dual anti-HER2 therapy before liver resection for rectal metastases

**DOI:** 10.1093/jscr/rjac420

**Published:** 2022-09-14

**Authors:** Siva Dantu, Asama Khan, Mashaal Dhir

**Affiliations:** SUNY Upstate Medical University, Department of Surgery, Syracuse, NY 13210, USA; SUNY Upstate Medical University, Department of Surgery, Syracuse, NY 13210, USA; SUNY Upstate Medical University, Department of Surgery, Syracuse, NY 13210, USA

## Abstract

This case presents a patient with a diagnosis of metastatic colon cancer on second-line chemotherapy. He demonstrated increased HER2 expression and was placed on dual HER2 antagonists. The patient had excellent repose and was ultimately able to undergo definitive liver resection for cure. We highlight the evolving use targeted therapy in metastatic colorectal cancer to not only extend progression-free survival, but act as a bridge to surgery.

## INTRODUCTION

Colorectal cancer (CRC) is the third most common cancer (estimated 151 030 new cases in 2022) and the second leading cause of cancer-related mortality (estimated 52 580 deaths in 2022) in the USA [1]. Approximately 25% of patients with CRC present with synchronous metastatic disease [2]. Around 60% of patients with CRC may develop liver metastases, while a subset of these patients will have liver-only metastatic disease. For patients with the unresectable liver-only disease, chemotherapy remains the backbone of treatment [3]. Although, first-line chemotherapeutic options are associated with median progression-free survival (PFS) of 9 months, median overall survival (OS) is longer (~2–3 years; [3]). Second-line chemotherapy regimens have a PFS of 3–6 months [4–6]. For patients with liver-only unresectable disease, there are several other options such as two stage hepatectomy, associating liver partition and portal vein ligation for staged hepatectomy (ALPPS), hepatic artery infusion chemotherapy, radioembolization and combination of surgery and ablation, which can be used in conjunction with systemic chemotherapy. Yet, surgical treatment options are limited for patients who are progressing on systemic therapy. Therefore, the addition of any effective systemic therapy allows more patients to become candidates for additional treatments including surgical treatments for CRC. We wanted to highlight and generate interest in one such treatment option in this case report.

## CASE REPORT

**Figure 1:**
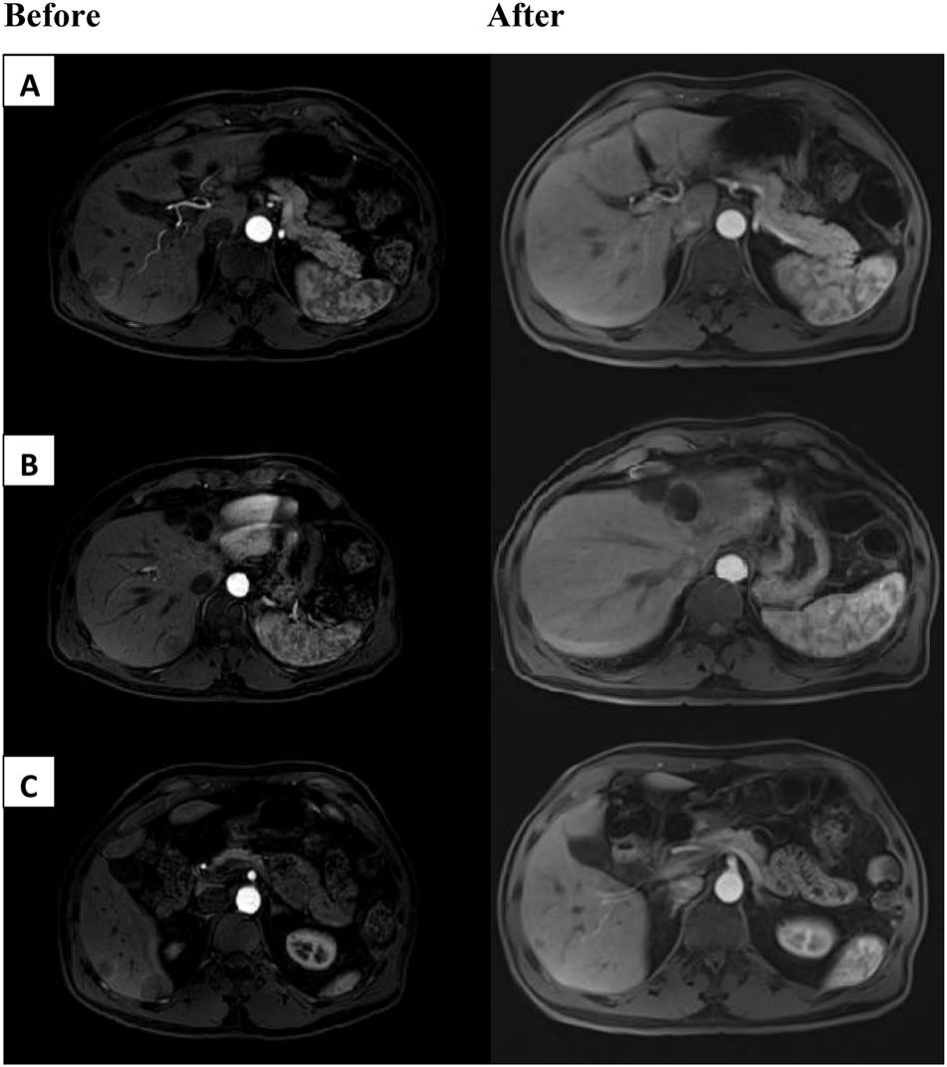
Above are representative images demonstrating severity of patient’s tumor burden, before (left) and after (right) dual anti-HER2 therapy. Patient had bilobar liver metastases ranging from 2.5 to 3 cm in segments 5, 7 and 8. Before images appear different than after due to institutional variation of MRI scanners.

We encountered a 67-year-old gentleman who was initially diagnosed with rectal cancer. This was determined to be 3 cm from the dentate line. MRI pelvis demonstrated T3N0 disease. The patient underwent neoadjuvant chemoradiation with capecitabine followed by low anterior resection with diverting loop ileostomy. The final pathology showed ypT3ypN0 disease. The patient deferred adjuvant chemotherapy. He subsequently underwent ileostomy takedown. The patient developed five liver lesions 2–3 cm in size on surveillance imaging 2 years later. He had a subsequent liver biopsy, which confirmed metastatic disease. The tumor was microsatellite stable. The CEA was 0.9 ng/ml at the time. The patient then underwent chemotherapy with FOLFOX and bevacizumab for 2 months. Unfortunately, he developed pancytopenia, poor appetite and functional decline on therapy. He was not felt to be a surgical candidate at this time. Additional testing on the tumor noted it to be Kirsten rat sarcoma virus (KRAS) wild-type (WT) and B-raf gene (BRAF) WT. The tumor was also noted to be 3+ for HER2 expression on immunohistochemistry (IHC). Given the decline in performance status and progression of the disease, he was then switched to dual Her2 antagonists, trastuzumab and pertuzumab therapy. The patient’s performance status improved and he tolerated the treatment without additional complications. The patient was then seen in hepatobiliary surgery clinic 4 months after initiation of trastuzumab and pertuzumab with interval MRI demonstrating marked reduction of metastatic disease (Fig. 1). After discussion in the multidisciplinary tumor board, the patient underwent definitive nonanatomic resection of the five different tumors. Pathology demonstrated ‘benign liver parenchyma with foci of fibrosis and inflammatory reaction consistent with treated metastasis’. This confirmed complete pathologic response in the patient after dual anti-HER2 therapy for metastatic rectal cancer. At the time of recent 1-year follow-up there was no evidence of recurrent disease.

## DISCUSSION

This case highlights the benefit of dual anti-HER2 therapy for patients with metastatic CRC. Although this approach has been used in the palliative setting, its role in the preoperative setting has not been described. It has been demonstrated that ~5% of patients with KRAS WT CRCs have a concomitant HER2 gene amplification [7]. HER2 antagonism has been the treatment of choice for metastatic breast cancer. In addition, anti-HER2 therapy has become the standard first-line treatment for gastric, esophageal, gastroesophageal junction tumors demonstrating HER2 gene amplification. The overall incidence of HER2 amplification in CRC ranges in the literature from 2 to 11% [8]. HER2 targeted therapy became a focus of research after preclinical models demonstrated increasing HER2 amplification in KRAS/BRAF WT cancers that had developed resistance to anti-EGFR therapy [9]. The HERACLES (Dual-targeted therapy with Trastuzumab and Lapatinib in treatment-refractory, KRAS codon 12/13 wild-type, HER2-positive metastatic colorectal cancer) trial in 2016 was the first phase II trial that investigated this subset of CRC patients. With a median follow-up of 94 weeks, it demonstrated a 30% objective response rate with one patient (5%) achieving a complete response after dual trastuzumab/lapatinib therapy in refractory metastatic CRC [10]. MyPathway is an ongoing phase II multiple basket study aimed at treatment-refractory solid tumors. One of the arms is the treatment of HER2 amplified tumors with pertuzumab with trastuzumab. The most recent subset analysis of patients with HER2 amplified metastatic CRC treated with trastuzumab and pertuzumab has demonstrated one patient (2%) with a complete pathologic response, and 17 patients (30%) with partial responses, with the median duration of the response being 5.9 months [11]. Most recently the Destiny-CRC01 trial investigated the use of Trastuzumab deruxtecan with an objective response rate of 45.3% after a median follow-up of 27.1 weeks [12]. Further studies to determine the relative efficacy of HER2-targeted therapy in comparison with more standard second-line agents have yet to be published.

Clinical trials so far have focused on using dual and single-agent targeted therapy as second and third-line regimens. Furthermore, the use of anti-HER-2 directed therapy with a chemotherapy backbone or single-agent conversion chemotherapy as a bridge to surgery has not been explored yet. If dual anti-HER2 therapy is being considered in the neoadjuvant setting, duration may have to be limited to less than 6 months given that median duration of response to HER2 blockade being 6 months in the trials. This case demonstrates potential for future research of dual anti-HER2 therapy in HER2 amplified CRCs in the neoadjuvant setting with intent to cure.

## CONFLICT OF INTEREST STATEMENT

There are no conflicts of interest involving the work under consideration for publication.

## FUNDING

This research did not receive any specific grant from funding agencies in the public, commercial or not-for-profit sectors.
